# Are the existing guidelines sufficient for the assessment of bathing water quality? The example of Polish lakes

**DOI:** 10.1007/s11356-021-13474-9

**Published:** 2021-03-24

**Authors:** Eugeniusz Pronin

**Affiliations:** Department of Plant Ecology, Faculty of Biology, University of Gdańsk, Wita Stwosza 59, 80-308 Gdańsk, Poland

**Keywords:** Bathing Water Directive, Harmful algal blooms, Quality of water, Water Framework Directive, Chlorophyll-*a*

## Abstract

The safety of beachgoers and swimmers is determined by the presence or absence of microbial contaminants and cyanobacterial toxins in the water. This study compared the assessment of bathing waters according to the Bathing Water Directive, which is based on the concentration of fecal contaminants, with some modifications, and a new method based on the concentration of chlorophyll-*a*, which corresponds to the World Health Organization (WHO) guidelines used for determining cyanobacterial density in the water posing threat to people health. The results obtained from the method based on chlorophyll-*a* concentration clearly showed that the number of bathing waters in Poland with sufficient and insufficient quality were higher in 2018 and 2019, compared to the method based on microbial contamination. The closing of bathing waters based only on the visual confirmation of cyanobacterial blooms might not be enough to prevent the threat to swimmers’ health. The multivariate analyses applied in this study seem to confirm that chlorophyll-*a* concentration with associated cyanobacterial density might serve as an additional parameter for assessing the quality of bathing waters, and in the case of small water reservoirs, might indirectly inform about the conditions and changes in water ecosystems.

**Supplementary Information:**

The online version contains supplementary material available at 10.1007/s11356-021-13474-9.

## Introduction

Lakes have many functions in the environment, from being a peculiar habitat for many unique species of plants and animals and thus increasing the local biodiversity (Declerck et al. 2006; Dudgeon et al. 2006; Downing 2010; Chester and Robson 2013; García-Girón et al. 2020), through water retention (Downing 2010; Nowak et al. 2018; Sterner et al. 2020), affecting the local climate, and utility (i.e., fishery) (Dudgeon et al. 2006) and recreational functions including bathing (Sender 2009; Dorevitch et al. 2011; Azevedo Lopes et al. 2016; Rosińska and Gołdyn 2018; Vierikko and Yli-Pelkonen 2019). In all the European Union (EU) countries, lakes with bathing waters are monitored according to the Bathing Water Directive (BWD) (EU 2006). Monitoring of bathing waters simply concerns microbial pollution (which includes only the indicators of fecal pollution such as *Escherichia coli* Escherich T. 1885 and intestinal enterococci concentration), based on which the quality of waters is determined as excellent, good, sufficient, or insufficient (EU 2006; Kataržytė et al. 2019). Additionally, lakes used for recreation may be monitored by the national monitoring systems, which take into account many water parameters indicated by the guidelines of the Water Framework Directive (WFD) (EU 2000). In Poland, this national system follows the WFD (EU 2000) and is called the State Monitoring System (SMS) which focuses on the assessment of the ecological status/potential primarily of water bodies having an area greater than 50 ha (Ciecierska and Kolada 2014).

Moreover, lakes used for bathing can be monitored through an assessment performed by the Natura 2000 system according to the guidelines of the Habitat Directive (HBD) (EU 1992). Based on the occurrence of characteristic plant species and the physical and chemical characteristics of waters, the system specified that the following habitats may offer a bathing space: 3110—lobelia lakes, 3140—charophytes lakes, 3150—eutrophic water reservoirs, and oxbow lakes. The assessment analyzes the conservation status of the lakes that are qualified to have the abovementioned habitats (Bolpagni et al. 2017; Kolada et al. 2017; Wilk-Woźniak et al. 2019). As shown by Bolpagni et al. (2017), some lakes in northern Italy are subjected to monitoring, simultaneously based on both WFD and HBD. Notably, similar monitoring is carried out in Poland but only in lakes larger than 50 ha, as shown in this study.

In Poland, bathing waters are not monitored for chlorophyll-*a* concentration; rather, monitoring involves only visual confirmation of the presence or absence of cyanobacterial blooms. However, the guidelines of the World Health Organization (WHO) concern the concentration of chlorophyll-*a* in the bathing waters as it is a proxy for cyanobacterial cells which may contain toxins capable of causing adverse health effects in beachgoers (Table 1) (WHO 2003; Kataržytė et al. 2019). According to these guidelines, a level of 10 μg/l chlorophyll-*a* with the dominance of cyanobacteria in the bathing waters is the threshold limit indicating protection from health outcomes due to the irritative or allergenic effects of cyanobacterial compounds. A level of 50 μg/l chlorophyll-*a* with the dominance of cyanobacteria indicates moderate cyanotoxin risk. The presence of cyanobacterial scum in swimming areas represents the highest risk of adverse health effects, as there is abundant evidence that potentially severe health outcomes are associated with cyanobacterial blooms (WHO 2003; Poniedziałek et al. 2012; Rzymski and Poniedziałek 2014). A study by Kokociński et al. (2013) showed that a detectable concentration of cyanobacterial cytotoxin cylindrospermopsin was found in almost 40% of 34 investigated lakes in western Poland during the summer period. Moreover, the above study was not the only one concerning toxin concentration in freshwater ecosystems in Poland. Kobos et al. (2013) reported that in 79% of 97 water bodies in Poland different toxins produced by cyanobacterial blooms were detected. Nevertheless, mentioned above the WHO guidelines are not implemented to the presented EU Directives. In Poland, national monitoring related to the WFD assumption involves only the assessment of chlorophyll-*a* concentration in large lakes (>50 ha) and large rivers, and transitional and coastal waters (Dz.U. 2019 poz 2149 2019). However, in the case of lakes, this information is now only a part of the calculated multimetric index for phytoplankton biomass and quality (Phytoplankton Metric for Polish Lakes (PMPL); Hutorowicz and Pasztaleniec 2014). Importantly, the WFD assumptions do not necessitate the assessment of chlorophyll-*a* concentration, and only recommend checking the biological quality elements such as phytoplankton biomass and diversity (EU 2000). This assessment might also be done based on chlorophyll-*a* concentrations, sometimes in combination with other variables such as nutrients concentrations (Primpas et al. 2010). Furthermore, the methodological guide for the monitoring of water habitats, such as lakes and oxbows, related to HBD in Poland recommends only the monitoring of phytoplankton with zooplankton as an auxiliary indicator for assessing the conservation of the monitored natural habitats under the Natura 2000 system (Mróz 2012, 2015; Kolada et al. 2017). In conclusion, in Poland, only for the lakes with an area greater than 50 ha, complex information about the lake condition might be available, if these lakes are included in the WFD and HBD monitoring. For lakes smaller than 50 ha, only HBD monitoring is performed if they have valuable habitats and are included in this monitoring system. Moreover, the condition of lakes, both smaller and larger than 50 ha, is partly determined and reflected by their biological parameters; for lakes larger than 50 ha, the Ecological State Macrophyte Index (ESMI), Diatom Index of Lakes (DIL), and PMPL can be listed. Other biological parameters, such as macroinvertebrates and fish, included in the evaluation of ecological state/potential, are also considered essential in Poland. However, the methodology used for the monitoring of macroinvertebrates sometimes provides inconclusive results (Moe et al. 2015) which are often excluded from the assessment of ecological state/potential (Chief Inspectorate of Environmental Protection (CIEP); https://www.gios.gov.pl/pl/stan-srodowiska/monitoring-wod) in Poland. In the case of small lakes, the macrophyte cover and structure, as well as phytoplankton structure, are also considered important biological variables influencing the water quality (Scheffer 1989; Scheffer and Van Nes 2007; Janssen et al. 2014; Wilk-Woźniak et al. 2019; Andersen et al. 2020).

**Table 1:** The WHO guidelines concerning chlorophyll-*a* concentration and the proposed implementation of WHO guidelines of chlorophyll-*a* concentration to BWD assessment schema of bathing water classification

The WHO guidelines concerning chlorophyll-*a* concentration	Bathing water status according to BWD additionally with "no data" category	The proposed classification for reassessment of BWD status depending on chlorophyll-*a* concentration
< 5 μg/l—no threat	Excellent	< 5 μg/l
from 5 μg/l to 10 μg/l—no threat	Good	5 μg/l to 10 μg/l
> 10 μg/l but < 50 μg/l irritative or allergenic effects of other cyanobacterial compounds (from 20,000 to 100,000 cyanobacterial cells/ml^−1^)	Sufficient	> 10 μg/l but < 50 μg/l
50 μg/l—moderate health alert in recreational waters, > 50 μg/l (more than 100,000 cyanobacterial cells/ml) and the presence of cyanobacterial scum in swimming areas represents the highest risk of adverse health effects	Insufficient	> 50 μg/l
–	Not evaluated	–
–	No data	not data

The report of the European Environment Agency (EEA 2019) states that the majority of both sea and inland bathing waters in EU were classified as having sufficient (in 2018—95.4%) and excellent quality (in 2018—85.1%) following the BWD classification. These situations, however, do not reflect the phenomenon of massive cyanobacterial blooms occurring more frequently due to climate changes (both in seas and inland waters, including lakes) (Paerl and Huisman 2008; Mantzouki et al. 2018; Huisman et al. 2018) and changes in the structures of aquatic vegetation, as well as in lakes with bathing waters in Poland (Pełechaty and Pełechata 2004; Pełechaty et al. 2006; Pukacz et al. 2007; Sender 2009; Pronin et al. 2011; Mikulski et al. 2017; Rosińska and Gołdyn 2018; Klimaszyk et al. 2020) in the light of other biological parameters, especially the concentrations of chlorophyll-*a*. Moreover, BWD monitoring considers only the microbiological contamination and visual confirmation of the presence or absence of cyanobacterial blooms which may not be informative enough for the assessment of health risk (Kataržytė et al. 2019).

Therefore, the main aim of this study (1) was to show that classification based only on fecal contamination is not appropriate to determine the quality of bathing waters, as it does not take into account the parameter reflecting the potential presence of cyanobacterial toxins in the water. For this purpose, bathing waters were classified based on chlorophyll-*a* concentration. It was assumed that most of the bathing waters would be identified as having insufficient and sufficient status when assessed according to the chlorophyll-*a* concentration, i.e., hazardous to humans, or where the risk of contact with cyanobacterial toxins is low, although they are acceptable—sufficient status. The second aim (2) was to check if there is a relationship between the status of bathing waters assessed based on chlorophyll-*a* concentration and microbiological contaminations (recorded in BWD monitoring) and the biological parameters included in the WFD monitoring. To achieve these aims, multivariate statistical analyses were applied.

## Materials and methods

### Study area

**Fig. 1:**
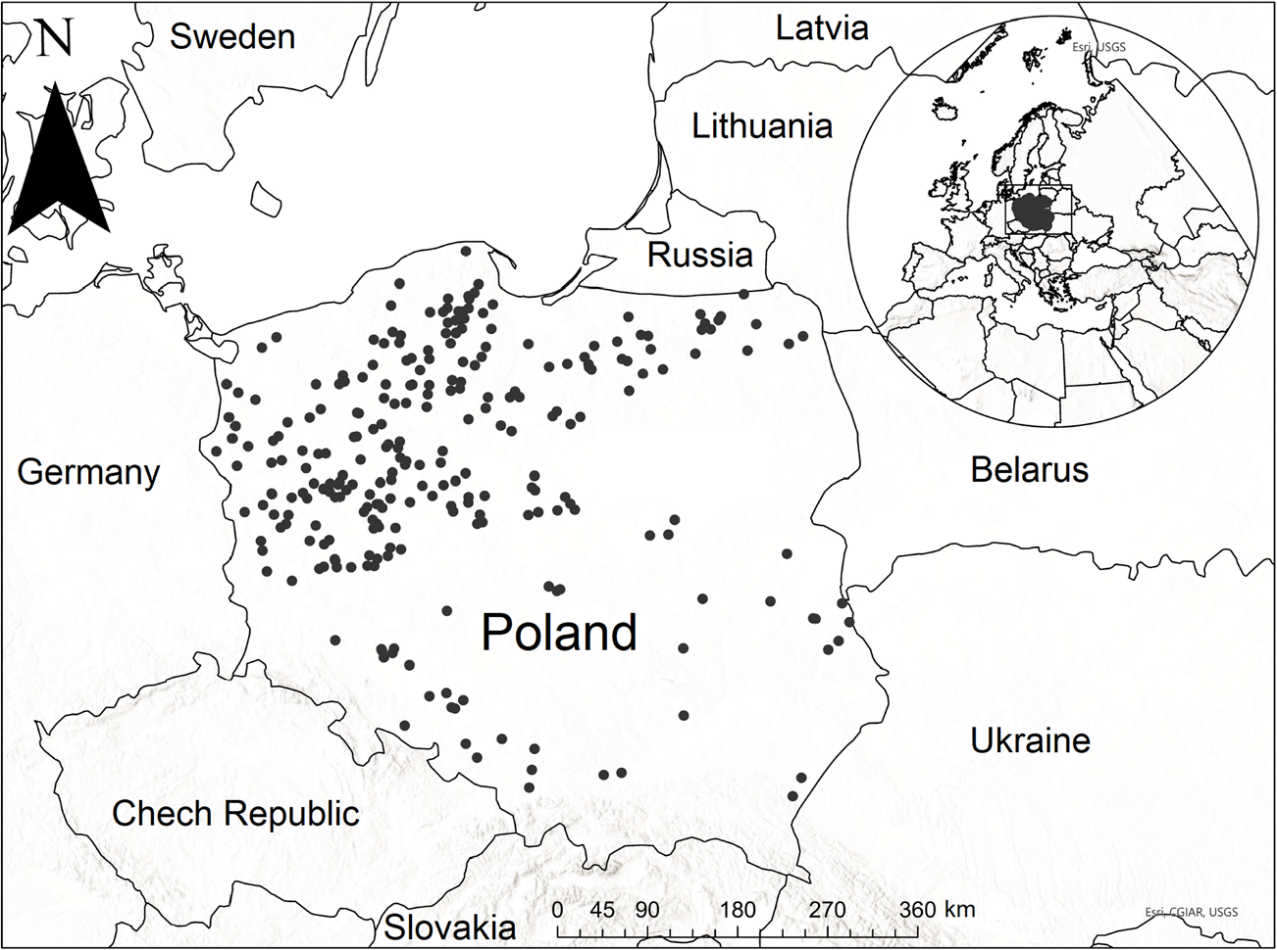
Localization of the bathing waters in Poland in 2019 bathing season

The study was performed on bathing waters, which were registered and monitored by the Chief Sanitary Inspectorate (CSI) in Poland in 2018 and 2019, mainly focusing on lakes, ponds, and water reservoirs (clay pit and gravel pit lakes). In Poland, a total number of 483 and 606 bathing waters were registered and monitored according to BWD in 2018 and 2019, respectively. There were 337 and 445 bathing places located in inland waters, of which 225 and 317 were on 196 and 268 water bodies, such as lakes, ponds, and water reservoirs (clay pit and gravel pit lakes), in 2018 and 2019, respectively (Fig. 1; Table S1 in Supplementary Materials 1) (bathing service: https://sk.gis.gov.pl/, accessed on 30.12.2020).

### Methods of data collection

Data for bathing waters were obtained from the materials published in the publicly available national bathing service website maintained by CSI. The data collected from the CSI report for 2018 and 2019 included the following: number of bathing waters, nature of the waters on which they were located, quality of bathing water based on BWD (i.e., 16 or 12 samples when the bathing season is no longer than 8 weeks); The data collected from own study based on the bathing service website maintained by CSI: number of lakes, bathing water surfaces of the analyzed lakes, quality of bathing water (for bathing waters, the number of available samples was not enough to perform the assessment according to BWD, and the assessment was based only on four samples, in conformity with the rule of 95 and 90 (when the status was in the range of insufficient) percentile (separately for 2018 and 2019)), and frequency of the occurrence of cyanobacterial blooms (status as of 30.12.2020). The data for water quality assessment for the category “lakes” were not available in the published reports (only data for the category “inland waters” were available). Therefore, based on CSI’s website database for bathing service, a database was prepared including data from water quality assessment based only on fecal pollutants from the years 2018 and 2019, respectively (Table S1 in Supplementary Materials 1). The data for bathing water assessment were available separately for particular lakes on the CSI bathing service website, and the list of lakes was specified for the 2019 bathing season. This is the reason why the numbers of lakes with bathing waters in 2018 and 2019 according to the prepared database were lower than the whole number of the lakes reported by CSI (2019, 2020). Additionally, the reports provided by CSI included the dam reservoirs under the category “lakes.” In this study, this type of water body was generally excluded because it is characterized by different ecology compared to the investigated lakes and ponds. The data for this work were collected after the bathing season in 2019 (bathing service: https://sk.gis.gov.pl/, accessed between 10.12.2019 and 30.12.2020).

Additionally, for a majority of lakes with an area of over 50 ha, the parameters taken into account in analyses were the ESMI, PMPL, DIL, chlorophyll-*a* concentrations (average for the whole growing season—usually average from four measurements), ecological status classification (based on the WFD restriction when the ecological state determined by the assessment of the worst parameter, usually PMPL associated with chlorophyll-*a* concentration). The results for the biological elements (ESMI, PMPL, DIL, and chlorophyll-*a* concentration) were obtained from published materials related to the monitoring of uniform parts of surface waters under the WFD conducted in Poland by the SMS coordinated by CIEP. The above data came from 2010 to 2018 because due to the 6-year monitoring cycle under the WFD in Poland, it was not possible to obtain results only for 2018 and 2019 for many of the investigated lakes. Furthermore, for bathing lakes, both greater and smaller than 50 ha, the available results of research related to the monitoring of aquatic habitats under the Natura 2000 network, also coordinated by the CIEP, were used. A literature review focusing mainly on the analyzed lakes was also performed (based on Google Scholar and Scopus databases accessed on 10.05.2020) (Table S1 in Supplementary Materials 1, e.g., Gołdyn 1991; Marszelewski and Noryśkiewicz 2003; Pukacz et al. 2007; Nędzarek and Tórz 2009; Napiórkowska-Krzebietke 2009; Pasztaleniec and Poniewozik 2010; Kaczorkiewicz 2010; Gołdyn et al. 2010; Goszczyński and Szatten 2013; Wiśniewska and Paczuska 2013; Kowalczewska-Madura et al. 2015; Dzieszko and Zwoliński 2015; Bryl et al. 2017; Tandyrak et al. 2017; Czerniawski and Krepski 2019; Osuch et al. 2020), from which chlorophyll-*a* values were collected for some lakes smaller than 50 ha. Besides, for two lakes smaller than 50 ha, the chlorophyll-*a* concentrations obtained from own study (using multiparameter device YSI 650 MDS with 6025 chlorophyll probe) (Table S1 in Supplementary Materials 1) conducted during 2020 were used. In addition, the chlorophyll-*a* concentration (usually the average was used if available) was obtained from the literature for several lakes larger than 50 ha when data about this parameter were not available in the results from SMS (Table S1 in Supplementary Materials 1). Chlorophyll-*a* values were used to evaluate the quality of bathing waters by referring to the indications contained in the WHO guidelines using the proposed BWD classification scheme (Table 1), in order to create a new factor that might be included for establishing the status of bathing waters. The proposed classification of bathing waters depending on chlorophyll-*a* concentration is shown in Table 1. The available data on chlorophyll-*a* concentrations for lakes greater (mainly from the data published by SMS) and smaller than 50 ha (literature data and own research carried out in 2020) were used to classify bathing waters according to the proposed classification. The “excellent” and “good” statuses were offered only to bathing waters of lakes where the chlorophyll-*a* concentration, according to the WHO guidelines, is not a threat to people (<10 μg/l). The concentration of chlorophyll-*a* was proposed not to exceed 5 μg/l to identify the lakes with perfect conditions. Additionally, the cyanobacterial density (individuals/ml) was calculated based on the chlorophyll-*a* concentration (calculation assumptions provided in Table 2 based on information from the WHO guidelines (WHO 2003) and Trophic State Index for lakes (Carlson 1977, 2007)).

**Table 2:** The established assumption for calculating the cyanobacterial density in investigated bathing waters depending on chlorophyll-*a* concentration values and assigned to Trophy State Index (Carlson 1977, 2007) and the four trophy state values based on chlorophyll-*a* concentrations. The multiplication factors correspond to cyanobacterial density values provided in WHO guidelines (WHO 2003)

The Carlson TSI classification based on the chlorophyll-*a* concentration with little modification of the values of the range of eutrophic and hypertrophic states	Chlorophyll-*a* concentration	Established cyanobacteria values depending on the trophic status of the water bodies	The percentage value of established values of cyanobacteria density depending on the trophic status of the water bodies corresponding 20 000 individuals per ml from in 10 μg/l chlorophyll-*a* concentration provided WHO guidelines
Oligotrophic	0–2.6 μg/l	2000 individuals/ml	10% of 20,000 individuals/ml
Mesotrophic	2.6–7.2 μg/l	4000 individuals/ml	20% of 20,000 individuals/ml
Eutrophic	7.2–50 μg/l	10,000 individuals/ml	50% of 20,000 individuals/ml
Hypertrophic	> 50 μg/l	20,000 individuals/ml	100% of 20,000 individuals/ml

### Statistical analyses

The normality of distributions of the analyzed variables was tested with the Shapiro–Wilk test using the Statistica 13.0 software (StatSoft Inc., Tulsa, OK, USA). The obtained results were not satisfactory; therefore, nonparametrical analyses were applied.

To test if the microbiological pollutants (*E*. *coli*, enterococci concentration, chlorophyll-*a* concentration, and calculated cyanobacterial density) corresponded to the assigned, new reassessed status of bathing waters based on the chlorophyll-*a* concentration, nonmetric multidimensional scaling (nMDS) was performed separately for 2018 and 2019 bathing seasons. This analysis was conducted in the R.4.0.3 software (R Core Team 2014) and the *vegan* package (Oksanen et al. 2019). For better visualization of the results of the nMDS analysis, the *ggplot2* package (Wickham 2009) of R was used. Additionally, the analysis of similarities (ANOSIM) test was performed to determine the similarity related to the performed nMDS analysis, using the *vegan* package (Oksanen et al. 2019). In both types of statistical analyses, similarity matrices were constructed using Bray–Curtis distance.

To test the relationships between the biological elements considered for the assessment of ecological status based on WFD monitoring and microbiological pollutants, the Spearman rank correlations were calculated and visualized using the plot as a heat map created in the *corrplot* R package. Finally, to test the detected correlations in relation to the investigated bathing water sites, a principal component analysis (PCA) was performed using the *FactoMineR* package (Lê et al. 2008) and the obtained results were visualized using the *factoextra* and *ggplot2* packages (Lê et al. 2008; Wickham 2009).

Prior to all the analyses, the data were log-transformed in the case of *E*. *coli*, enterococci density, chlorophyll-*a* concentration, and cyanobacterial density to avoid scale effects. The ESMI, PMPL, and DIL indices were excluded in this log-transformation, as the matrices were constructed in the range from 0 to 1 in the case of ESMI and DIL and from 0 to 5 for PMPL.

## Results

In Poland, 483 bathing sites located in the Baltic Sea and inland waters were reported in 2018, while 606 bathing waters were registered in 2019 (Fig. S1 in Supplementary Materials 2). A rapid increase in the number of bathing waters was observed in Poland since 2018, which continued in 2019. The CSI reports (CSI 2019, 2020) provided data about all categories of bathing waters in Poland. This study investigated a total of 225 bathing waters in 2018 and 317 bathing waters in 2019 located on lakes and other water bodies included in this category (Table S1 in Supplementary Materials 1).

**Fig. 2:**
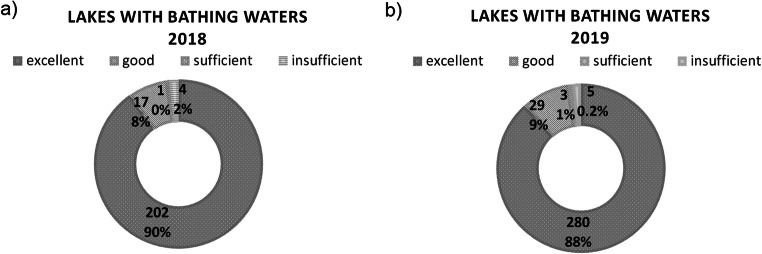
The number of bathing waters in Poland with the BWD classification of the water quality with the addition of the own classification based only on the fecal pollutants data from particularity bathing season: **a** bathing waters during the bathing season in 2018, **b** bathing waters during the bathing season in 2019 (based on the bathing service website provided by CSI: https://sk.gis.gov.pl/ status as of 30.12.2020)

The quality assessment of all bathing waters based on fecal pollutants showed that waters with excellent status dominated, especially in 2018. Additionally, bathing waters classified as sufficient and insufficient accounted for about 2% in both the investigated bathing seasons (Fig. 2a, b).

**Fig. 3:**
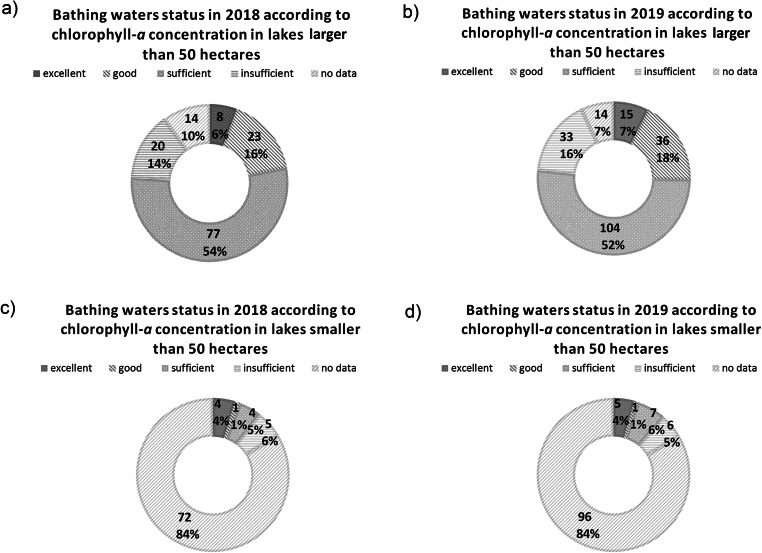
Distribution of bathing waters assessed status on lakes in 2018 and 2019 based on chlorophyll-*a* concentration in the bathing waters (classification according to the assumptions provided in Table 1). The results are divided into two groups of lakes: **a** and **b** status classification based on chlorophyll-*a* concentration in bathing waters located in lakes larger than 50 ha in 2018 and 2019, respectively; **c** and **d** status classification based on chlorophyll-*a* concentration in bathing waters located in lakes smaller than 50 ha in 2018 and 2019, respectively (own study based on the database provided in SMS: access of 10.05.2020, supplemented with data found in the literature and own research conducted in July 2020; the literature and own data are presented in Table S1 in Supplementary Materials 1)

The quality assessment of bathing waters based on chlorophyll-*a* concentration (Table 1) carried out separately for lakes greater and smaller than 50 ha showed different results compared to those based only on the density of microbiological pollutants (Fig. 2, Fig. 3). Notably, in the group of larger lakes, the majority of the bathing waters were characterized by chlorophyll-*a* concentration in the range of 10–50 μg/l (Table S1 in Supplementary Materials 1). A chlorophyll-*a* concentration above 50 μg/l was recorded in 36 (17%) lakes larger than 50 ha (Table S1 in Supplementary Materials 1). In smaller lakes, for the majority of water bodies, no data on chlorophyll-*a* concentration were available.

Evaluation based on chlorophyll-*a* concentration showed that more lakes were included in the group with insufficient water levels both in 2018 and 2019 compared to the evaluation based on microbiological pollutants (according to BWD) (Fig. 3). The number of reevaluated lakes with insufficient status was higher among lakes larger than 50 ha. The bathing sites in these lakes most commonly were of sufficient status, both in 2018 and 2019 (Fig. 3). However, the opposite trend was observed when this classification was compared to the microbiological assessment (according to BWD regulations) shown previously indicating the most abundant group was bathing waters with excellent status (Fig. 2). However, for bathing waters in lakes smaller than 50 ha, the group “no data” dominated (Fig. 3b, d).

**Fig. 4:**
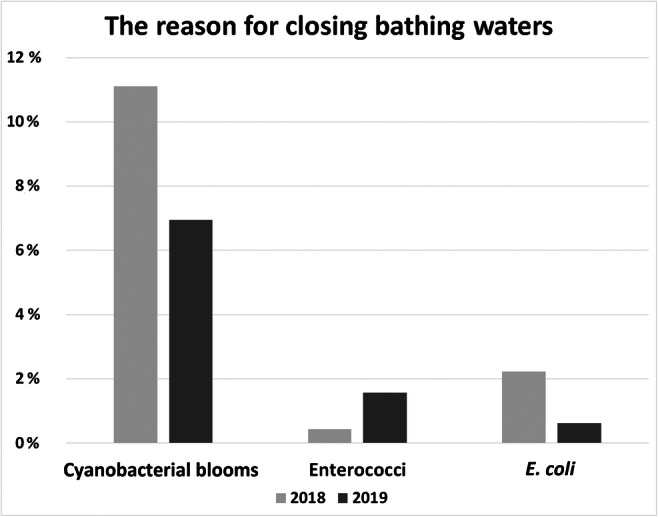
The percentage of bathing waters with cyanobacteria blooms, *E*. *coli* and enterococci concentration caused the closing of the lakes bathing water for bathers at least once in Poland’s bathing season. Data was shown for bathing seasons 2018 and 2019 (data for 2018, and 2019 own study based bathing service website provided by CSI: https://sk.gis.gov.pl/https://sk.gis.gov.pl/ status as of 30.12.2020)

Cyanobacterial blooms, which are one of the critical factors in the BWD monitoring of bathing waters in Poland, were observed more often in 2018 bathing season than in 2019. For lakes in 2018, these blooms caused the closing of 25 bathing waters at least once, and in 2019 22 lakes were closed (Table S1 in Supplementary Materials 1, Table S2 in Supplementary Materials 2). Moreover, cyanobacterial blooms were noted in bathing waters characterized mostly by sufficient and insufficient status based on chlorophyll-*a* concentration (Table S2 in Supplementary Materials 2), determined as 11.15% and 6.9%, respectively (Fig. 4). Microbial pollutants caused closing of bathing waters relatively less often than cyanobacterial blooms, but closing was still observed (Fig. 4). Both 2018 and 2019 seasons had more significant numbers of occasional events of bathing site closing compared to the previous years (Fig. S1 in Supplementary Materials 2).

**Fig. 5:**
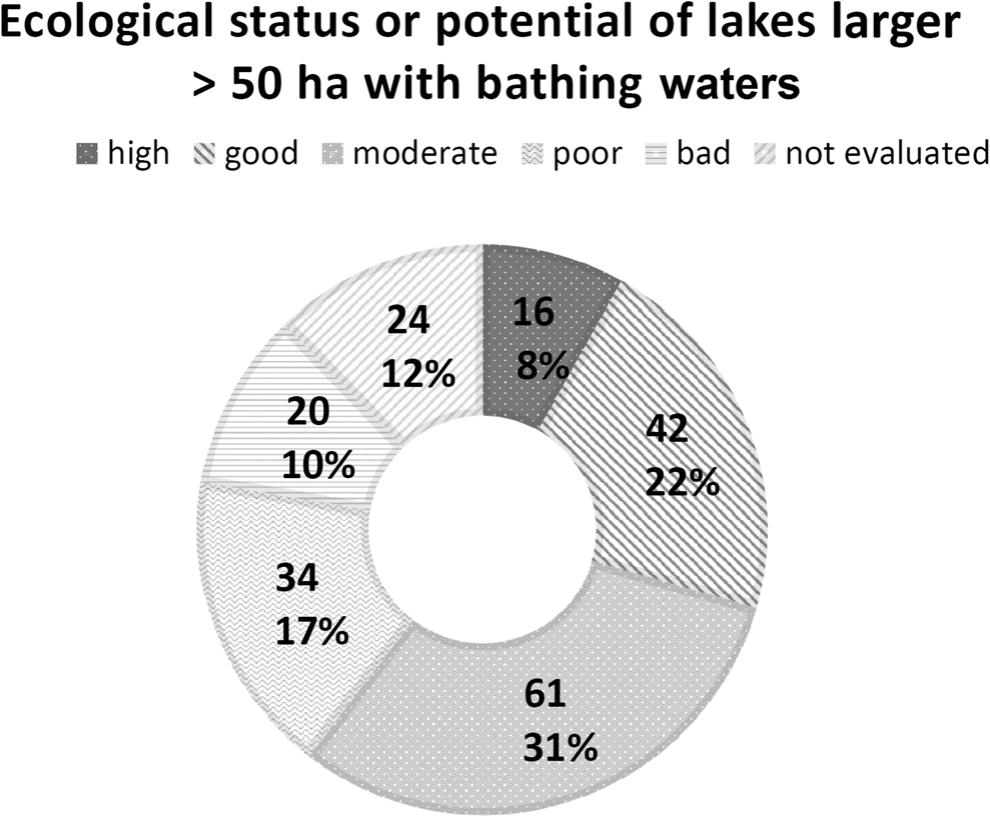
Ecological status or potential of lakes > 50 ha with bathing waters in 2019 (own study based on the database provided in SMS: access of 10.05.2020—the extrapolated assessment without the results of biological parameters was not included; it was assigned to the category not evaluated)

Additionally, for lakes greater than 50 ha which were included in the ecological state/potential assessment of SMS (Fig. 5; Table S1 in Supplementary Materials 1), a comparison of these assessments was made. This group contained 24 bathing waters on larger lakes where SMS was not performed (Fig. 5). However, only the assessment of ecological status/potential was extrapolated based on other information such as anthropological stress and the type of lake catchment (data not shown). The ecological status/potential of the majority of bathing waters was found to be classified as moderate and good, followed by poor and bad (Fig. 5).

**Fig. 6:**
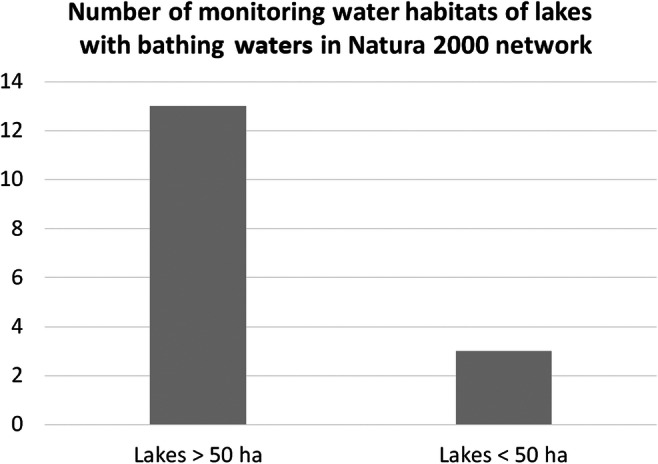
The number of monitoring water habitats of lakes with bathing waters in Natura 2000 network (own study based on the database provided by CIEP reports: status as of 10.05.2020)

The available data from the CIEP reports on monitoring by the Natura 2000 network showed that lakes greater than 50 ha were more often included in this protection system than the smaller lakes (Fig. 6). The number of habitats in larger lakes was four times greater than lakes smaller than 50 ha (Fig. 6; Table S1 in Supplementary Materials 1).

**Fig. 7:**
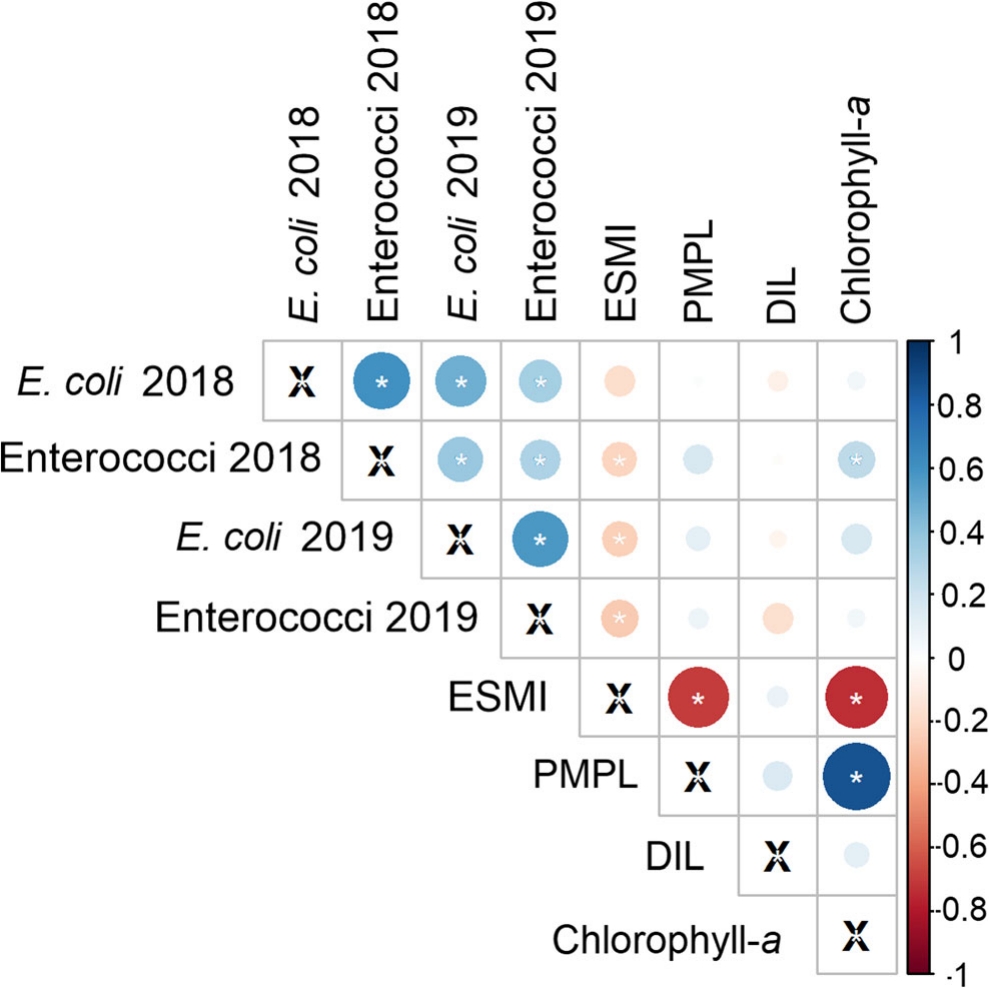
The matrix of Spearman rank correlations presented as a heat map of the relationships between the biological elements of ecological status assessment based on WFD monitoring and microbiological pollutants. The * indicated the statistical significance in the *p* < 0.05 level. *N* =317 between *E*. *coli* and enterococci in 2019, *N* = 225 between *E*. *coli* and enterococci in 2018, *N* =142 for ESMI in 2019 and *N* = 94 in 2018, *N* = 175 in 2019 and *N* = 116 in 2018 for PMPL, *N* = 91 for DIL in 2019 and *N* = 63 in 2018, *N* = 207 in 2019 and *N* = 142 in 2018 for chlorophyll-*a* concentration (own study based on the database provided in SMS coordinated by CIEP: status as of 10.05.2020 and on the website database provided by CSI in the bathing service https://sk.gis.gov.pl/ status as of 30.12.2020)

The correlation between the biological parameters of the ecological status/potential assessment and data on microbiological pollutants was analyzed (the Spearman rank correlation). A strong negative and statistically significant relationship was found between ESMI and chlorophyll-*a* concentration, as well as PMPL, which is determined partly based on chlorophyll-*a* concentration. A strong positive relationship between PMPL and chlorophyll-*a* concentration confirmed this association (Fig. 7). Moreover, a weak negative but statistically significant correlation was found between *E*. *coli* (2019) and enterococci density (2018 and 2019) and ESMI. Additionally, strong and weak positive relationships were observed between microbiological pollutant density in both investigated bathing seasons (Fig. 7). Notably, cyanobacterial density calculated based on the assumptions provided in Table 2 showed an identical correlation matrix as chlorophyll-*a* concentration and thus was not included in this analysis.

**Fig. 8:**
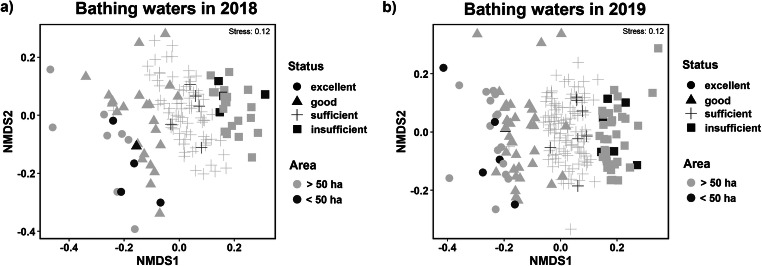
The ordination diagrams of nonmetric multidimensional scaling (nMDS) analysis for the data matrix when the bathing waters were considered as sites (rows) and microbiological contamination (*E*. *coli*, and enterococci density, chlorophyll-*a* concentration, and cyanobacteria density) as a species (columns) for **a** bathing waters in 2018 and **b** for bathing waters in 2019. The symbols represent the reassessed status based on the chlorophyll-*a* concentration: circle—excellent; triangle—good; cross—sufficient; square—insufficient; the light-gray color corresponds to the bathing waters in lakes > 50 ha, and the black-gray color corresponds to the bathing waters in lakes < 50 ha. *N* = 142 for 2018 and *N* = 207 for 2019

**Fig. 9:**
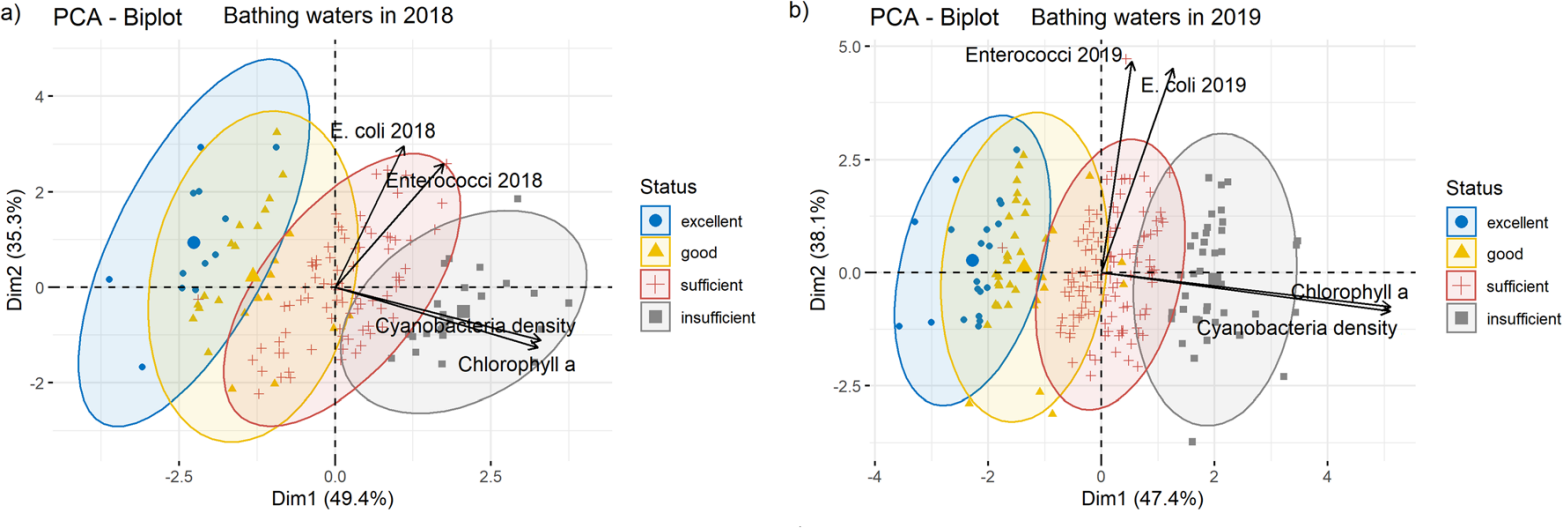
The ordination diagrams of principal component analysis (PCA) for investigated bathing waters and microbiological contaminations performed for **a** bathing waters in 2018, **b** bathing waters in 2018. *N* = 142 for 2018 and *N* = 207 for 2019. The ellipses means 95% concentrations of the sites in the selected groups. The symbols represent the status based on the chlorophyll-*a* concentration. *N* = 142 for 2018 and *N* = 207 for 2019

The nMDS analysis performed to check how all the microbiological pollutants (*E*. *coli*, enterococci, cyanobacterial density, and chlorophyll-*a* concentration) corresponded to the assigned, new status of bathing waters based on chlorophyll-*a* concentration clearly showed that in 2019 the new evaluation method of bathing waters corresponded well to microbial contamination (Fig. 8a). A similar situation was noted in 2018 (Fig. 8b). Generally, only a few bathing waters did not correspond to the new assigned status, as shown in Fig. 8. However, most of them were placed in the correct group, especially in the 2019 bathing season (Fig. 8b). The ANOSIM, which was performed to supplement the presented nMDS plots, showed that in both 2018 and 2019 the status of the assessed group of bathing waters was significantly different (ANOSIM statistic: *R* = 0.34, *p* = 0.0001 and *R* = 0.59, *p* = 0.0001 in 2018 and 2019, respectively). Moreover, the ANOSIM comparing between lakes larger and smaller than 50 ha showed that these groups did not differ much (ANOSIM statistic: *R* = 0.07, *p* < 0.1736 and *R* = 0.10, *p* = 0.0582 in 2018 and 2019, respectively). Additionally, to analyze how the microbiological contaminations were distributed and the investigated variables corresponded to each other, a PCA was performed separately for the 2018 and 2019 bathing season (Fig. 9a, b). For 2018, the first two main components explained 84.7% of the total variance and for 2019 they explained 85.5%. A clear distinction was observed between the groups assigned to the respective bathing water status in both investigated seasons. However, similar to the nMDS analyses, several bathing waters were located in other groups, or some of the excellent, good, and sufficient status groups overlapped each other. Similarly, in 2018 and 2019, the cyanobacterial density was strictly related to chlorophyll-*a* concentration, and these were correlated with the first main component. In addition, *E*. *coli* and enterococci density were strongly related to each other and were correlated with the second main component (Fig. 9a, b).

**Fig. 10:**
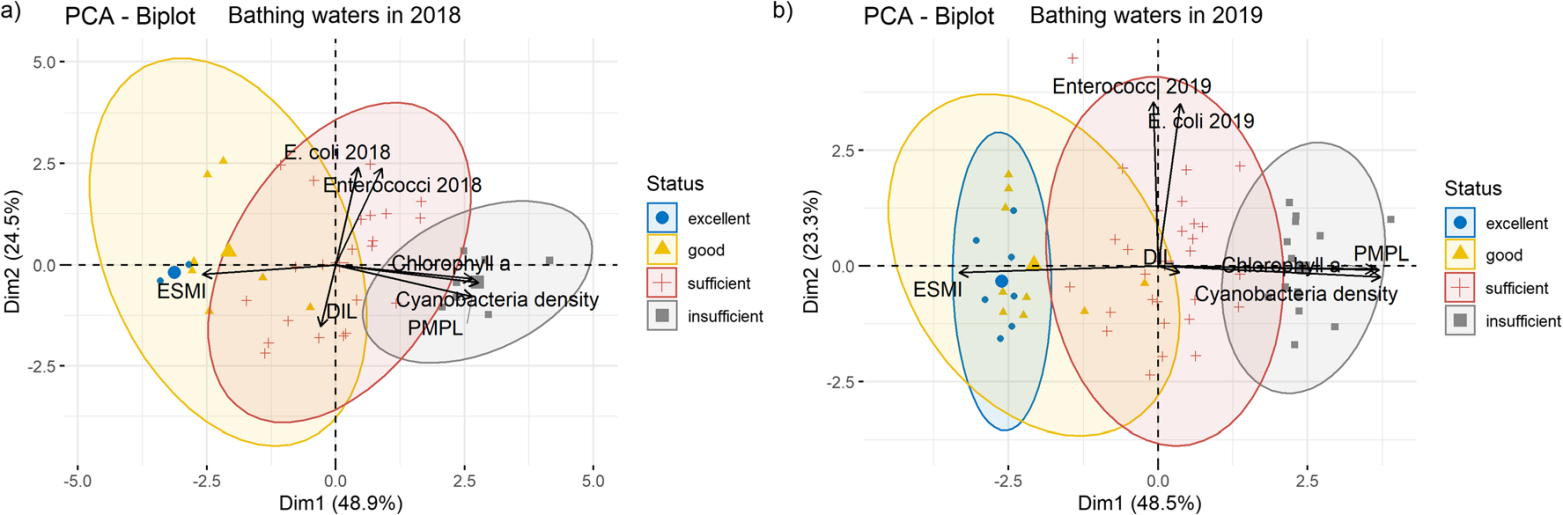
The ordination diagrams of principal component analysis (PCA) for investigated bathing waters in relation to microbiological contaminations and biological parameters controlled in WFD monitoring in Poland in lakes > 50 ha, **a** bathing waters in 2018, **b** bathing waters in 2018. The ellipses means 95% concentrations of the sites in the selected groups. The symbols represent the status based on the chlorophyll-*a* concentration. *N* = 63 for 2018 and *N* = 91 for 2019

Finally, to compare the Spearman rank correlations and the distribution of microbiological contaminations, a PCA for bathing waters where all these parameters were available was performed. In 2018, a lower number of bathing waters were assigned to the excellent status where all other parameters were available (Fig. 10a); thus, the ellipse with 95% concentrations was not calculated. Other status assessments of bathing waters were divided by PCA into separate clusters. The two main components of PCA explained 73.4% of the total variance in 2018 and 71.8% in 2019. In 2019, more data were available; thus, all four clusters corresponding to bathing water status were divided by PCA (Fig. 10b). Notably, the separated clusters overlapped, especially in the case of excellent and good assessment status of bathing waters (Fig. 10a, b) which corresponded to the previously presented nMDS analyses. In both 2018 and 2019, the same pattern was observed where the chlorophyll-*a* concentration, PMPL, and cyanobacterial density were strongly related to each other and negatively correlated with ESMI. These four variables were associated with the first principal component axis. Furthermore, in both investigated years, *E*. *coli* and enterococci density were strongly related to each other, and were associated with the second principal component axis (Fig. 10a, b).

## Discussion

### The issue of cyanobacterial blooms of the bathing waters

The observed temperature increase in the globe is one of the main reasons for people using more often the aquatic ecosystems for recreation including bathing (Vierikko and Yli-Pelkonen 2019). In Poland, about a 75% increase in bathing waters was noted between the 2018 and 2019 bathing seasons. This increase in Poland was not only related to the demand for ecosystem services of the local population due to the increasing mean temperature (especially in the summer period) but also due to changes in the law regulations related to bathing waters which encouraged the local administration, as well as private owners of water bodies, to register the bathing waters. However, climate change significantly affect the water bodies, providing optimal conditions for the occurrence of cyanobacterial blooms which are noticed more often (Paerl and Huisman 2008; Huisman et al. 2018; Mantzouki et al. 2018; Kataržytė et al. 2019; Chen et al. 2020; Overlingė et al. 2020). Cyanobacterial blooms, as shown in this study, were the main reason for the closing of many bathing waters (Fig. 3, Fig. S1 in Supplementary Materials 2; Table S1 in Supplementary Materials 1) to protect people from potentially toxic effects (WHO 2003; Poniedziałek et al. 2012; Rzymski and Poniedziałek 2014; Kataržytė et al. 2019; Overlingė et al. 2020). However, as mentioned in the “Introduction” section, bathing waters are assessed only by microbiological contamination. Notably, this is not the only threat to beachgoers and swimmers, and the harmful toxins produced by cyanobacterial blooms might be present in the water not only during the visual confirmation of blooms but even before and after the confirmation (Kobos et al. 2013).

Moreover, according to BWD, to evaluate particular bathing waters to determine the assessment procedure, continuous monitoring must be conducted for four consecutive bathing seasons (16 valid samples are required or 12 in special circumstances, for example, if the bathing water is opened shorter than 8 weeks or situated in a region subject to special geographical constraints) (EU 2006). If these requirements are not met, the status of the bathing water is considered not assessed (EU 2006). Thus, for this study, two types of evaluations were carried out on bathing waters: (1) one based on microbiological contamination, according to BWD with modification; and (2) second based only on the concentration of chlorophyll-*a*. The chlorophyll-*a* concentration might be a better predictor of the presence of cyanobacterial toxins compared to the visual confirmation of cyanobacterial blooms, especially when it is measured in time intervals during the whole bathing season as fecal contaminations. The occurrence of blooms does not affect the classification of bathing waters into one of the four listed categories (excellent, good, sufficient, insufficient), and the presence of blooms is the only descriptive character in the evaluation process of the bathing water assessment of BWD. Thus, the country reporting the bathing water quality to EEA does not have an obligation to provide this information. Due to this reason, it is hard to find details about past and recent cyanobacterial blooms in the bathing waters considered for this study. Many European countries, including Poland, develop national websites that can be used for checking the recent and sometimes past assessment of bathing water quality. Unfortunately, a majority of them, including the bathing website in Poland, do not have an English version, which might decrease their accessibility to visitors from other countries (the list of the national or regional pages related to BWD is presented on the EEA website).

Although many countries in the world prepared and have been using the protocols and guides related to cyanobacterial blooms, most of them have not implemented the national regulations (Ibelings et al. 2014). The actions taken for bathing water assessment related to cyanobacterial blooms differ in countries over the world, including Poland (Table 2 in Chorus 2012). According to the BWD in Poland, water samples for microbiological analysis must be taken no less than four times during the bathing season. (In Poland, the bathing season starts on 1.06 and ends on 30.09 which means at least one analysis is done per month—usually, it is more often due to the individual agreement and monitoring schedule settled between the administrator of the bathing water and CSI. Furthermore, in BWD, the upper limit of sampling through the bathing season is not fixed). Moreover, during sampling, an observation of the presence of cyanobacteria must be carried out (Chorus 2012). This observation is also an obligatory task in the everyday protocol of the administrator of bathing water before the water is opened to beachgoers and swimmers. However, more restricted guidelines and regulations were created, for example, in the Netherlands, New Zealand, and some other countries where the concentration of chlorophyll-*a* and even that of toxins is determined (Table 2 in Chorus 2012). Ibelings et al. (2014) showed that in several EU countries, after the new regulation of WFD and BWD, the national institutions dropped the projected plans of developing new, more restricted protocols including the assessment of cyanotoxin risk related to cyanobacterial blooms, scums, and mats present in the waters. Thus, the change of the evaluation method of bathing waters, as presented in this study, in which chlorophyll-*a* concentration was added (according to the assumptions presented in Table 1), caused an increase in the number of lakes with worse status assessment, than when the current and applicable methodology was used. Similar findings were presented by Kataržytė et al. (2019) who also stressed the lack of assessment of harmful algal blooms (HAB) in the BWD. The results of multivariate analyses, such as nMDS and PCA (in which the relationship between the cyanobacterial density and chlorophyll-*a* concentration, as well as microbiological contaminants included in BWD, was tested), presented in this study confirmed that the BWD methodology should be adapted to evaluate the threats posed by climate change, i.e., increase of cyanobacterial blooms and potential occurrence of toxins in water, and their impact on human health.

Moreover, the study and assessment of HAB in the Baltic Sea in the Lithuania region confirmed the presence of cyanotoxins (Overlingė et al. 2020). Furthermore, as was mentioned in the “Introduction” section, the study by Kokociński et al. (2013) showed that cyanobacterial cytotoxin (cylindrospermopsin) was present in almost 40% of 34 investigated lakes in western Poland. Similarly, the study by Kobos et al. (2013) showed that in 79% of 97 water bodies, including several lakes with bathing waters (Table S1 in Supplementary Materials 1), different toxins produced by cyanobacterial blooms were detected among which microcystins (96%) dominated. These cyanobacterial toxins might have adverse effect e.g., cylindrospermopsin and microcystins include hepatotoxicity, genotoxicity, dermatotoxicity, and fetal toxicity on human health (Poniedziałek et al. 2012; Kobos et al. 2013). These findings are in line with those of another study performed in Germany, in which the authors showed that cytotoxin was detected in 50% of 127 German lakes (Fastner et al. 2007). Taking into account the obtained results, Overlingė et al. (2020) recommend routine monitoring of cyanotoxins in the south-eastern Baltic Sea during the bathing season. Thus, the level of potentially toxic cyanobacteria in bathing waters (especially in the Baltic Sea) systematically increases, and this issue should also be addressed in other water reservoirs due to the increasing average global temperature.

### The issue of monitoring

Small reservoirs are the sources of key ecosystem services, including bathing sites (Kristensen and Globevnik 2014). The presented studies have shown that small water bodies with bathing waters were significantly less frequently monitored in all the referred monitoring programs. The reason for this in Poland is the law regulation requiring that only lakes larger than 50 ha might be monitored in the national SMS system coordinated by CIEP (Ciecierska and Kolada 2014; Dondajewska et al. 2019). Thus, small lakes, ponds, and other water reservoirs used for bathing did not have a chance to be investigated in detail, unlike the larger lakes, in accordance with the WFD in EU countries. Only microbiological monitoring and water surface observation are performed during the monitoring of bathing waters located in small lakes (EU 2006). When the lakes were included in the Natura 2000 habitat network, one could relatively rarely find more information about the water parameters and biological variables. In lakes investigated according to the WFD implemented in Poland (Ciecierska and Kolada 2014), biotic and abiotic analyses are more complicated. This situation also proves that in the EU regulation, there is more focus on larger lakes than the smaller ones. Large lakes might be considered as more permanent ecosystems and might provide more ecosystem services, primarily related to recreation (Ziv et al. 2016; Vierikko and Yli-Pelkonen 2019; Sterner et al. 2020). However, it should be remembered that in many European countries, and also in Poland, small water bodies constitute the vast majority of inland water reservoirs (Kristensen and Globevnik 2014) and thus should also be monitored to some extent (especially when the presence of cyanobacteria is noticed in the bathing waters). As Kristensen and Globevnik (2014) mentioned in their study, some of the EU countries included smaller water bodies in the WFD-related monitoring systems when the water bodies were protected under other legislation or if they were ecologically important in the river basin. In addition to monitoring under BWD, the presented study emphasizes the importance of research carried out as part of scientific works of local and cognitive nature. These scientific works are essential and sometimes the only source of knowledge about a given water body.

In this study, it was confirmed that negative correlations exist between the calculated macrophyte index (ESMI), which is a good predictor of the macrophyte condition in the lakes (Ciecierska and Kolada 2014), chlorophyll-*a* concentration, which is more or less reflected by the nutrient conditions in the water bodies (Jordan et al. 1991; James et al. 2009; Liang et al. 2020), and PMPL, which is strictly associated with the chlorophyll-*a* concentration. However, in order to have more reliable conclusions, these relationships should be considered taking into account the morphometric difference between the lakes. Studies from the last few decades have confirmed the alternative stable state theory in shallow lakes (Scheffer 1989; Scheffer and Van Nes 2007; Janssen et al. 2014; Andersen et al. 2020). The authors of those studies showed that in deep and shallow lakes, the ecological response to additional nutrient load is different and is usually more rapid in the case of shallow and small lakes than deep and large ones. Thus, small and shallow lakes, as well as other small water bodies, are more sensitive to the anthropogenic input associated with recreational usage (not only bathing waters) and also to changes in their catchment (Davies et al. 2009; Boix et al. 2012; Kuczyńska-Kippen and Pronin 2018) which might be reflected by vegetation changes in such lakes (Pełechaty and Pełechata 2004; Pronin et al. 2011; Chmara et al. 2015; Rosińska and Gołdyn 2018). However, in larger lakes, changes especially of the sensitive type of macrophytes, which form isoetide groups of plants in soft water lakes, might be noticed, and as the authors conclude; these changes were observed due to increasing recreational use of lakes, as well as the changes in the direct catchment (Klimaszyk et al. 2020). The next group of aquatic plants that are sensitive to the environmental changes of water and also to changes in the catchment area, as well as increasing recreation pressure, are charophytes (Królikowska 1997; Bociąg et al. 2011; Krupska et al. 2012; Pukacz et al. 2013; Pełechaty et al. 2014, 2015; Pronin et al. 2016, 2018).

Thus, considering the higher sensitivity of small water bodies to changes in the catchment and the additional load of nutrients resulting from the recreational activities of people, small water bodies with bathing waters in particular should be additionally monitored with the obligatory assessment of chlorophyll-*a* concentration. This monitoring might be supported or even performed by the remote sensing methods to minimize the costs of the chlorophyll-a analysis as many authors were already suggested (Kataržytė et al. 2019; Chen et al. 2020; Overlingė et al. 2020). As pointed above, small water bodies are especially characterized by rapid changes and the water quality might transition from good (“the clear water state”) to bad (“the turbid water state”) (Scheffer 1989; Scheffer and Van Nes 2007; Janssen et al. 2014; Andersen et al. 2020). Those rapid changes might threaten the beachgoers and swimmers due to toxins originating from cyanobacteria present in water. The toxins might not be identified only by visible confirmation of cyanobacterial blooms because they might be present even if the blooms are no longer seen (Kobos et al. 2013).

The arguments presented here, as well as the results of this study, indicate the need to take actions for the development of some monitoring systems or inclusion of additional parameters (especially obligatory checking of chlorophyll-*a* concentrations, e.g., using remote sensing methods) for monitoring of bathing waters according to BWD. Complex monitoring of water bodies with bathing waters will provide more information to better protect the health of bathing water users and better manage and implement appropriate restoration procedures.

## Conclusion

The quality of bathing waters, and therefore, the usefulness of aquatic ecosystems for bathing, varies greatly depending on the type of ecosystem. The lakes and other water bodies with bathing waters are often monitored, but as pointed in this study monitoring related to BWD does not seem to be appreciative. This is particularly true in the case of small lakes and water bodies used for bathing purposes where only microbiological contamination are investigated and visual confirmation of the presence or absence of cyanobacterial blooms is carried out. As highlighted in this study, there is a lack of obligatory checking of cyanotoxin and chlorophyll-*a* concentration in the BWD. Inclusion of these parameters in the routine procedure of the assessment of bathing water status should be considered.

## Supplementary Information

ESM 1 (XLSX 49 kb).

ESM 2 (DOCX 156 kb).

## Data Availability

Most data generated or analyzed during this study are included in this article and its supplementary materials files. The rest of the included data are available from the author on reasonable request.
