# Supplementary material for: Are the existing guidelines sufficient for the assessment of bathing water quality? The example of Polish lakes
Source: Environ Sci Pollut Res Int. 2021 Mar 24;28(29):39742–56. doi: 10.1007/s11356-021-13474-9 (PMC8310518; doi:10.1007/s11356-021-13474-9)
Supplement: Supplementary file 2 — (DOCX 156 kb). [file 11356_2021_13474_MOESM2_ESM.docx]

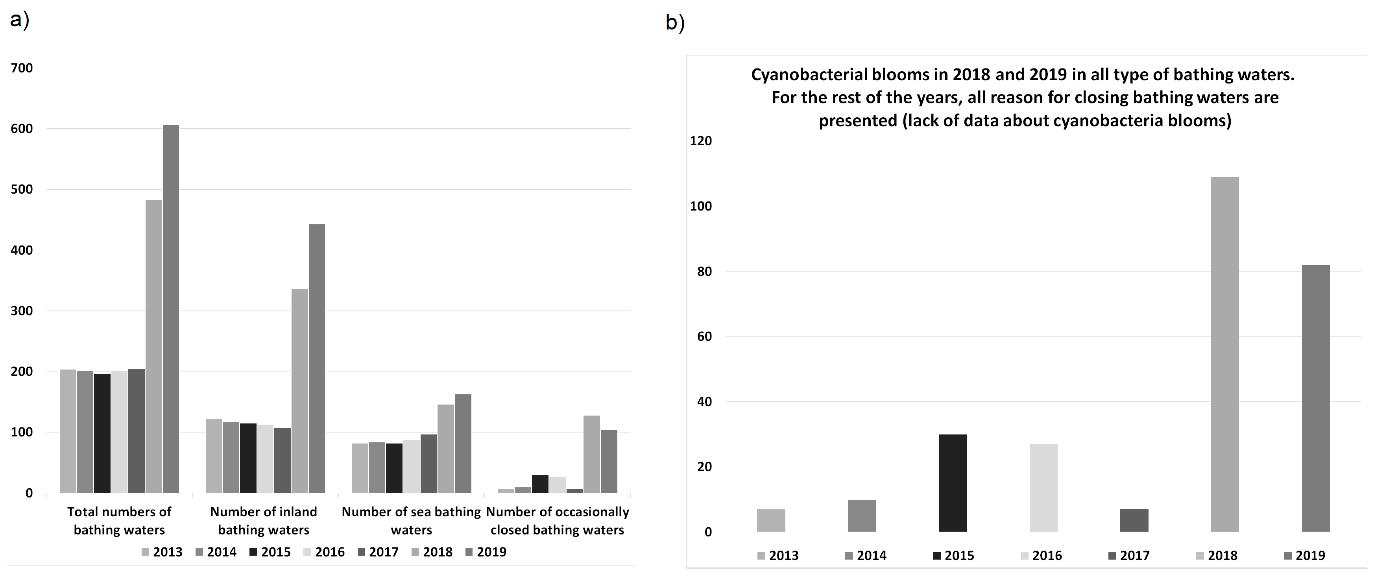


Fig. S1 The number of the bathing waters in Poland from 2013 to 2019 where a) the total number, distribution of the bathing waters in different types of waters (Baltic Sea and inland waters) and number of occasionally closed bathing waters in particular years, b) cyanobacterial blooms in all types of bathing waters for 2018 and 2019 in three light to previously years where the total number of occasionally closing water was presented due to not provided information about the cyanobacterial blooms.

Table S2. The reassessed bathing water status based on the chlorophyll-*a* concentration in bathing waters which were occasionally closed because of the cyanobacterial blooms.

| Bathing season | Cyanobacteria blooms | Excellent status | Good status | Sufficient status | Insufficient status | n.d. | n.d. in lakes below 50 ha |
| --- | --- | --- | --- | --- | --- | --- | --- |
| 2018 | 25 | 2 | 1 | 7 | 4 | 10 | 8 |
| 2019 | 22 | 0 | 3 | 8 | 3 | 8 | 6 |

Table S3. The Wilcoxon test for comparing the differences between chlorophyll-*a* concentration and calculated cyanobacterial density with or without division into lakes larger than 50 ha and lakes smaller than 50 ha in 2018 and 2019

| Group of samples | N | Z | P |
| --- | --- | --- | --- |
| 2018 all samples | 142 | 10.34 | 0.001 |
| 2018 larger than 50 ha | 128 | 9.82 | 0.001 |
| 2018 smaller than 50 ha | 14 | 3.30 | 0.001 |
| 2019 all samples | 207 | 12.47 | 0.001 |
| 2019 larger than 50 ha | 188 | 11.89 | 0.001 |
| 2019 smaller than 50 ha | 19 | 3.82 | 0.001 |
